# A Label-Free Fluorescent AND Logic Gate Aptasensor for Sensitive ATP Detection

**DOI:** 10.3390/s18103281

**Published:** 2018-09-29

**Authors:** Jingjing Zhang, Chunzheng Yang, Chaoqun Niu, Chen Liu, Xuepin Cai, Jie Du, Yong Chen

**Affiliations:** State Key Laboratory of Marine Resource Utilization in South China Sea, College of Information Science & Technology, College of Materials and Chemical Engineering, Institute of Tropical Agriculture and Forestry, Hainan University, Haikou 570228, China; zhangjingjingaoxue@163.com (J.Z.); chunzhengyang@163.com (C.Y.); chaoqunniu@163.com (C.N.); chen__liu@126.com (C.L.); 13698954926@163.com (X.C.)

**Keywords:** ATP detection, label-free fluorescence, enzyme-free, graphene oxide, logic gate

## Abstract

In this study, a label-free fluorescent, enzyme-free, simple, highly sensitive AND logic gate aptasensor was developed for the detection of adenosine triphosphate (ATP). Double-stranded deoxyribonucleic acid (DNA) with cohesive ends was attached to graphene oxide (GO) to form an aptasensor probe. ATP and single-stranded DNA were used as input signals. Fluorescence intensity of PicoGreen dye was used as an output signal. The biosensor-related performances, including the logic gate construction, reaction time, linearity, sensitivity, and specificity, were investigated and the results showed that an AND logic gate was successfully constructed. The ATP detection range was found to be 20 to 400 nM (R^2^ = 0.9943) with limit of detection (LOD) of 142.6 pM, and the sensitivity range was 1.846 × 10^6^ to 2.988 × 10^6^ M^−1^. This method for the detection of ATP has the characteristics of being simple, low cost, and highly sensitive.

## 1. Introduction

Adenosine triphosphate (ATP) is a direct source of energy in the body and has a great influence on biological processes [1,2]. Many scholars have shown that the concentration of ATP is closely related to the health of the human body. For example, low concentrations of ATP can affect the metabolic activity of organisms, resulting in biological cell damage and loss of vitality [3,4], and high concentrations of ATP may cause hypoxia, hypoglycemia, and Parkinson’s disease [5]. Therefore, it is very necessary to develop a quantitative detection method for ATP.

Fluorescent biosensors have been widely used to detect substances such as ions, nucleic acids, organic molecules, and proteins [6,7,8]. There are two common methods for improving the sensitivity of deoxyribonucleic acid (DNA) fluorescence sensors, which are: increasing the output signal or suppressing the background signal. Common methods for signal amplification include strand displacement amplification [9,10,11,12], rolling circle replication [13], and the use of DNA enzymes [14,15,16,17], etc. However, although these methods can effectively increase the sensitivity of a sensor, they usually suffer from large background signal and require the use of biological enzymes. Enzymes are easily affected by reaction temperature and pH and are relatively expensive to use.

Compared with signal amplification, the background signal suppression method can lead to the design of a sensor with a simpler structure. The inhibition of the background signal can be achieved using graphene oxide (GO) [18,19,20], gold nanoparticles [21,22], magnetic nanoparticles [23], two-dimensional sheet metal-organic frameworks [24] as a quencher, which increases the sensitivity of the sensor. However, these materials usually involve the use of DNA with fluorescent labels, resulting in the detection method being relatively high cost. Therefore, label-free fluorescence methods are favored by researchers. Dyes, such as PicoGreen [25,26], thiazole orange (TO) [27], and SYBR Green I [28], intercalate in double-stranded DNA to produce fluorescence, but do not fluoresce in single strands of DNA. Thioflavin T dye forms an G-quadruplex complex with the G-rich aptamer and results in high fluorescence signal [29,30]. These dyes have the advantages of being simple, rapid to use, and low in cost. However, the disadvantage of the dyes is that they do not use signal amplification or suppression of the background signal to increase the sensitivity of the sensor. After a literature search, no reports were found on the detection of ATP using a simultaneous label-free, enzyme-free, and background signal inhibition method.

The application of molecular computers in medicine, nanotechnology, and biotechnology is very significant. Molecular logic gates form the hardware basis of molecular computers and can perform various molecular Boolean operations. Up to now, many logic gates have been constructed, such as AND, INH+NINH, and INHIBIT [19,31,32]. Although many molecular logic gates have been established, there is still a need to construct logic gates that are simpler, more sensitive, and are lower in cost. For these reasons, in this work, GO and PicoGreen dye were used to construct biosensors that have a simple structure for highly sensitive, label-free fluorescence, and enzyme-free ATP detection. It is hoped that the general method developed in this work can be adopted for the detection of various different proteins and ions.

## 2. Materials and Methods

### 2.1. Reagents and Materials

ATP, cytidine triphosphate (CTP), uridine triphosphate (UTP), guanosine triphosphate (GTP), and PicoGreen dye were purchased from Shanghai Yi Sheng Technology Co., Ltd. (Shanghai, China). All oligonucleotides were supplied by Beijing Genomics Institute (Beijing, China). The sequences of DNA-T, DNA-M, and DNA-M’ are listed in detail in Table 1. PicoGreen dsDNA quantitation reagent was purchased from Shanghai Yi Sheng Biotechnology Co., Ltd. (Shanghai, China). Graphene oxide sol (graphene oxide content: 1 wt%) was purchased from Shanghai Aladdin Biochemical Technology Co., Ltd. (Shanghai, China). Other reagents were purchased from Beijing Lian Shi Yun Shang Network Technology Co., Ltd. (Bejing, China).

### 2.2. The Process Used for Detection

All reagent dilutions were performed using a buffer (10 mmol/L Tris, 50 mmol/L NaCl, 10 mmol/L MgCl_2_, pH 7.5 and ultradeionization). 1 µM DNA-T and 1 µM DNA-M were mixed and the resulting mixture was then heated to 95 °C for 5 min. Subsequently the mixture was cooled to room temperature in a furnace before finally being stored in a refrigerator at 4 °C. DNA-M′ was also heated to 95 °C for 5 min, and was then placed in a refrigerator at about −20 °C for 5 min, and was then finally stored at 4 °C in the refrigerator. the PicoGreen dye was diluted to 200-fold by buffer. The GO was diluted to 100-fold by buffer. In all of the experiments, the total amount of buffer used was 1825 µL, the 1 µM DNA-T and DNA-M mixture totaled 88 µL, the amount of 1 µM DNA-M′ used was 90 µL, the spectral detection of PicoGreen dye was achieved using 40 µL of dye, the reaction time of the detection of the PicoGreen dye was achieved using 30 µL of dye, and a total of 10 µL of GO was used. Before each experiment, the buffer and reagent were placed in a 2 mL sample tube, and the reaction mixture was kept for at least 3 h at room temperature.

### 2.3. The Selectivity Assays

The blank sensing system was made up of 1825 µL buffer, DNA-T + DNA-M mixture totaled 88 µL, 90 µL DNA-M’, 10 µL GO and 40 µL PicoGreen dye. The ATP sensing system consisted of the blank sensing system and 200 µL of 10 µM ATP. The UTP sensing system consisted of the blank sensing system and 200 µL of 10 µM UTP. The CTP sensing system consisted of the blank sensing system and 200 µL of 10 µM CTP. The GTP sensing system consisted of the blank sensing system and 200 µL of 10 µM GTP. The mixture sensing system was made up of the blank sensing system, 200 µL of 10 µM ATP, 200 µL of 10 µM UTP, 200 µL of 10 µM CTP, and 200 µL of 10 µM GTP. After the sensing systems incubating for 3 h, fluorescence intensity was tested by the proposed method.

### 2.4. ATP Detection in Real Urine Samples

A healthy adult urine was obtained from Hainan University hospital (Haikou, China). The urine samples were centrifuged at 13,000 r/min for 3 min. The resulting supernatants were subsequently diluted to 10-fold with buffer solution and the pH of the resulting solutions was 7.5. ATP was added in diluted urine at concentrations of 100, 300 and 500 nM, respectively, repeating the measurement three times. The recovery was then computed.

### 2.5. Fluorescence Spectroscopy

The change in the fluorescence intensity of the sensing liquid in a 10 mm square quartz cuvette was recorded using a fluorescence spectrometer (RF-6000, Shimadzu, Tokyo, Japan) with magnetic stirring. The spectral detection parameters used were an emission wavelength of 480 nm and a scanning range of 495 nm to 700 nm. The reaction time program detection parameters used were an emission wavelength of 480 nm and an excitation wavelength of 520 nm.

## 3. Results and Discussion

### 3.1. Construction of an AND Logic Gate

**Figure 1:**
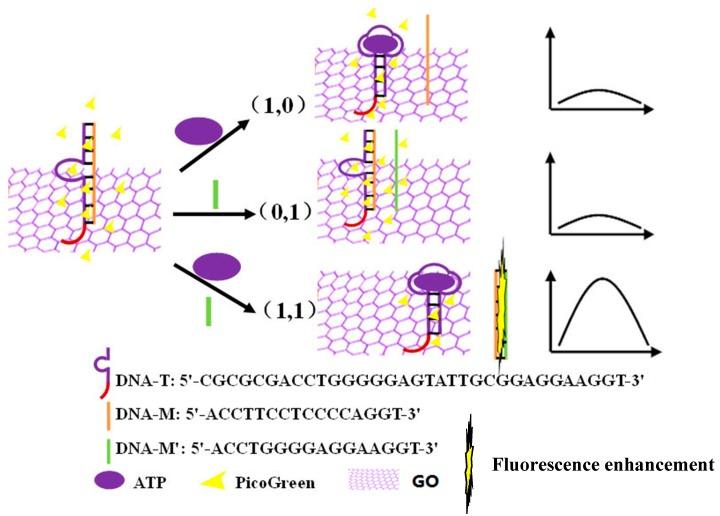
The principle of AND logic gate construction.

ATP and DNA-M′ were used as input signals, and the fluorescence intensity change of PicoGreen was used as an output signal to construct an AND logic gate, a schematic overview of which is shown in Figure 1. The input signal was 1 if ATP or DNA-M′ were present, if not, it was 0. Meanwhile, at a wavelength of 527 nm, if the PicoGreen fluorescence intensity increased, the output signal was 1, if not, the output signal was 0. As shown in Figure 1, DNA-T and DNA-M double strands were used as a sensor probe and GO was used to inhibit the background signal. If both ATP and DNA-M′ were not present, the input signal was (0,0). The DNA-T and DNA-M double strands adsorbed on the surface of GO through π-π stacking leading to significant fluorescence quenching and an output signal of 0. In the absence of ATP or DNA-M′, the input signal was (0,1) or (1,0). All of the DNA was found to adsorb on GO and the fluorescence of the probe was obviously quenched and the output signal was 0. However, when ATP and DNA-M′ were introduced into the system, the input signal was (1,1). The binding of ATP and a DNA-T chain led to the release of DNA-M. DNA-M and DNA-M′ combined to form double-stranded DNA, which was released from the surface of GO due to the weaker affinity between the double strands and GO. PicoGreen dye was embedded in the double-stranded DNA and the fluorescence was significantly enhanced as a result, with an output signal of 1.

**Figure 2:**
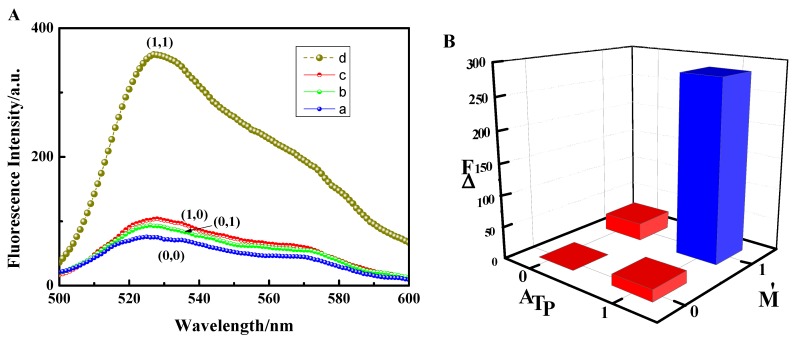
(**A**) Fluorescence emission spectra under different conditions: (a) DNA-T, DNA-M, and GO; (b) DNA-T, DNA-M, GO, and DNA-M’; (c) DNA-T, DNA-M, GO, and ATP; (d) DNA-T, DNA-M, GO, DNA-M’, and ATP. (**B**) Logical histogram.

In order to verify the feasibility of the AND logic gate, spectral analysis was performed, as shown in Figure 2A. The a-curve in Figure 2A shows that in the absence of both ATP and DNA-M′, the fluorescence intensity at 527 nm (the following fluorescence values are all at 527 nm) was 75.28 a.u. When double-stranded DNA with sticky ends adsorbed onto graphene oxide through π-π stacking, a lower fluorescence value was observed due to fluorescence resonance energy transfer (FRET). The b-curve in Figure 2A shows the fluorescence intensity value of 92.82 a.u. in the presence of DNA-M′ only. Since the DNA-M’ base and the DNA-T base fragment are identical, DNA-M′ could not open the DNA duplex (DNA-T + DNA-M′). The sticky-ended DNA double-strands and DNA-M′ single-strands all adsorbed onto the GO, resulting in the occurrence of FRET, which led to lower fluorescence values being observed. The c-curve in Figure 2A shows that the fluorescence intensity value was only 103.20 a.u. in the presence of ATP. ATP can open DNA double strands (DNA-T + DNA-M′), to form hairpins with sticky ends, resulting in the release of DNA-M. All of the DNA was adsorbed on GO, which led to the occurrence of FRET, resulting in poor fluorescence. However, in the presence of both ATP and DNA-M′, the fluorescence intensity was 359.05 a.u., as shown by the d-curve, also in Figure 2A. Due to ATP being bound to DNA-T, DNA-M was released. DNA-M′ formed a perfectly complementary double strand with DNA-M, which was then released from the surface of GO due to the weaker affinity between the double strand and GO. PicoGreen dye embedded in the double strand and produced relatively strong fluorescence [25]. A comparison of the d-curve with the other three curves (the a-, b-, and c-curves) showed that the fluorescence intensity was significantly increased in the presence of both ATP and DNA-M′.

**Figure 3:**
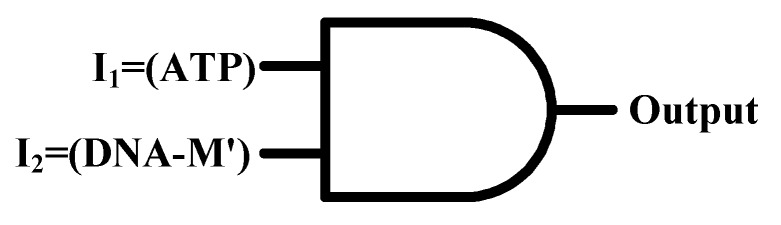
Schematic of an AND logic gate.

To further construct the logic gate, ATP and DNA-M′ were used as input signals. When they were present, the input signal was 1, otherwise it was 0. The differences between the peaks of the four curves in Figure 2A and the peak of the a-curve were taken as the output signal, respectively. If the fluorescence intensity difference, ΔF, was greater than 30 a.u., the output signal was 1, otherwise it was 0. The results are shown in Figure 2B and Table 2. Figure 2B shows that the output was 0 when the inputs were (0,0), (0,1), and (1,0). However, when the input was (1,1), the output was 1, which matches Boolean AND logic. Therefore, using the above method, an AND logic gate was constructed and a schematic representation of the AND logic gate is shown in Figure 3.

### 3.2. An Investigation into the Reaction Time

**Figure 4:**
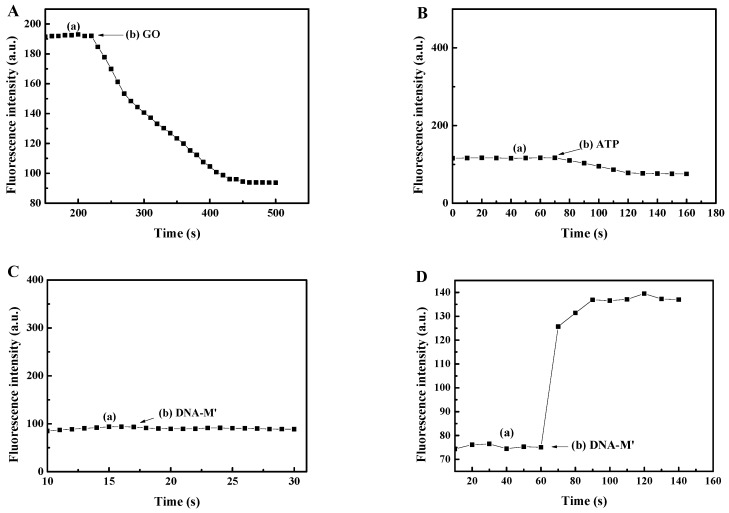
The investigation of the reaction time: (**A**) (a) DNA-T and DNA-M, (b) GO; (**B**) (a) DNA-T and DNA-M, (b) 200 µL of 10 µM ATP; (**C**) (a) DNA-T, DNA-M, and GO, (b) DNA-M′; (**D**) (a) DNA-T, DNA-M, 200 µL of 10 µM ATP and GO, (b) DNA-M′.

To further verify the feasibility of the principle, timed detection was carried out, as shown in Figure 4. Figure 4A shows that the fluorescence intensity rapidly decreased when GO was added. It was demonstrated that the GO has the effect of suppressing the background signal, which is consistent with what has been described in the literature [18,19] and the a-curve shown in Figure 2A. As shown in Figure 4B,C, the fluorescence intensity was found to slightly change after adding ATP or DNA-M’. Due to all of the DNA being adsorbed on the surface of GO through π-π stacking, FRET occurred, resulting in a lower fluorescence value. This was illustrated by the fact that the fluorescence intensity was relatively weak when only ATP or DNA-M′ was present as an input, which further validated the b-curve and c-curve data shown in Figure 2A. Figure 4D shows that the fluorescence intensity rapidly increased when the DNA-M′ single strands were added. As DNA-M′ rapidly formed a completely complementary DNA duplex with DNA-M, it detached from the surface of the GO. The PicoGreen dye intercalated into the double-stranded DNA, leading to the generation of strong fluorescence. This proved that the fluorescence intensity became stronger when ATP and DNA-M′ were simultaneously used as inputs, which also verified the d-curve data shown in Figure 2A. Therefore, it can be concluded that Figure 4 is consistent with what has been described in Figure 2A and the principle is feasible.

### 3.3. Analysis of the Linearity and Sensitivity

**Figure 5:**
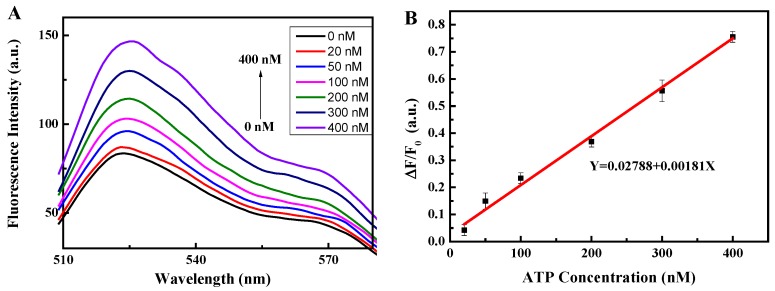

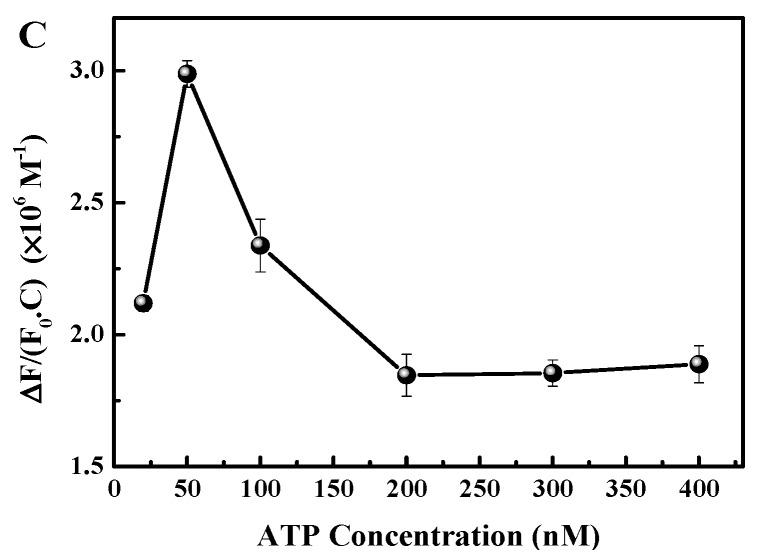
(**A**) Spectral detection at different ATP concentrations; (**B**) the linearity of the sensor; (**C**) the sensor sensitivity. DNA-T, DNA-M, DNA-M’, GO, and ATP (0 nM, 20 nM, 50 nM, 100 nM, 200 nM, 300 nM, 400 nM).

The 0 nM ATP curve shown in Figure 5A was used as the initial value, F_0_, and the other curve peaks were used as the final F values. The linearity is shown in Figure 5B. The linear correlation coefficient R^2^ was found to be 0.9943, which proves that the sensor has very high linearity. The linear equation was Y = 0.0278 + 0.00181X and the measurement range was found to be 20–400 nM. In this linear range, the concentration of target ATP can be estimated quantitatively using an equation. The limit of detection (LOD) was 142.6 pM (3σ/slope). Compared with the most of the other literatures (Table 3), this sensor has higher linearity and smaller detection limit. Therefore, it can be concluded that this method is very suitable for the detection of ATP over this linear range.

Figure 5C shows that the sensitivity first increased and then decreased. The sensitivity when 50 nM of ATP was used was found to be higher than that using 20 nM of ATP. One possible reason for this was that 50 nM of ATP opened up more double strands (DNA-T + DNA-M) of DNA and released more DNA-M. DNA-M′ and DNA-M were then able to form completely complementary double strands and detach from the GO surface. PicoGreen dye embedded in the double-stranded DNA, resulting in strong fluorescence. Thus, the relative change rate of the fluorescence, ΔF/F_0_, of 50 nM of ATP was greater than that of 20 nM of ATP, as shown in Figure 5B. In addition, the difference between the 50 nM ATP and 20 nM ATP concentration was small. From the sensitivity formula ΔF/(F_0_·C), it can be seen that the 20 nM ATP sensitivity was lower than that of the 50 nM ATP sensitivity. From Figure 5B, it can also be seen that the relative change rates of ATP for 100, 200, 300 and 400 nM were higher than that of 50 nM of ATP, but their concentrations were much greater than 50 nM. Therefore, from the sensitivity formula ΔF/(F_0_·C), it can be deduced that their sensitivity is less than that of the 50 nM of ATP. The sensitivity range of the sensor was found to be 1.846 × 10^6^ to 2.988 × 10^6^ M^−1^, as shown in Figure 5C. It can therefore be concluded that this method is very sensitive for detecting ATP.

### 3.4. Analysis of the Selectivity

**Figure 6:**
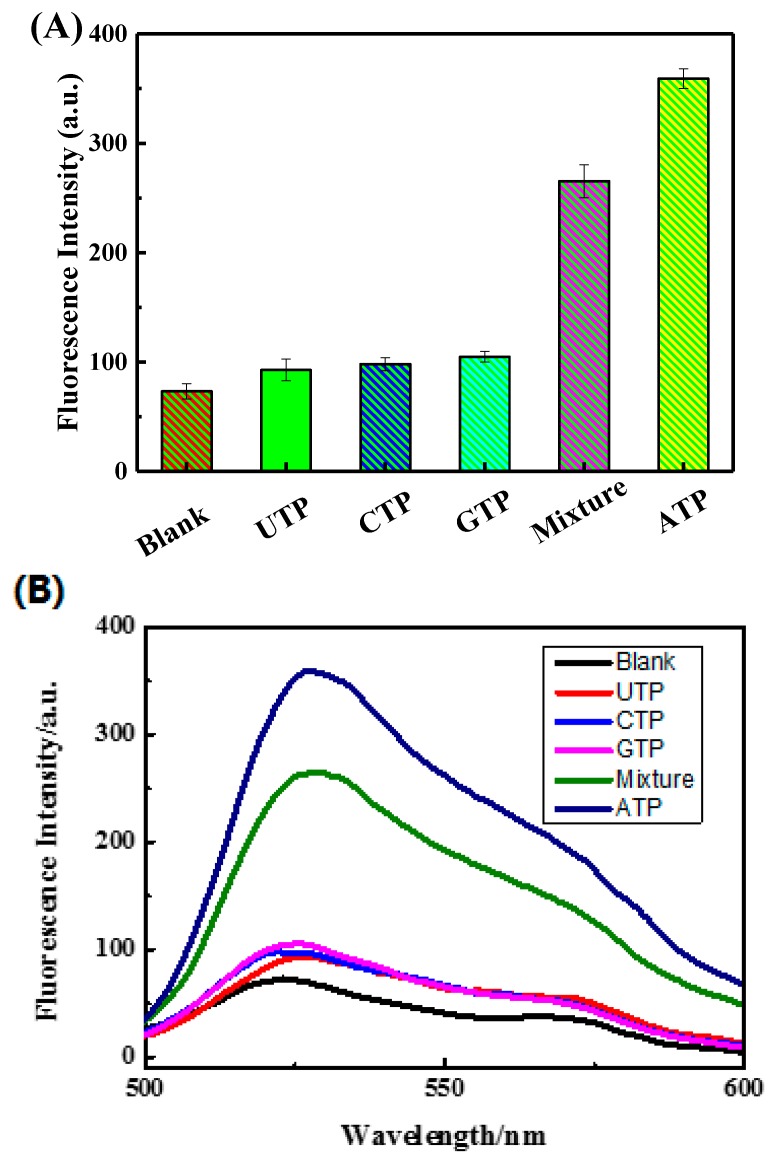
(**A**) The selectivity assays. Blank sensing system consisted of DNA-T, DNA-M, DNA-M′, GO and PicoGreen dye; (**B**) ATP sensing system consisted of the blank sensing system and 200 µL of 10 µM ATP; UTP sensing system consisted of the blank sensing system and 200 µL of 10 µM UTP; CTP sensing system consisted of the blank sensing system and 200 µL of 10 µM CTP; GTP sensing system consisted of the blank sensing system and 200 µL of 10 µM GTP; Mixture sensing system consisted of the blank sensing system, 200 µL of 10 µM ATP, 200 µL of 10 µM UTP, 200 µL of 10 µM CTP, and 200 µL of 10 µM GTP.

Figure 6 shows that the fluorescence intensity of the blank sensing system was relatively weak because the blank sensing system didn’t involve ATP. The fluorescence intensities of UTP, CTP, and GTP sensing system were found to be similar to the background fluorescence due to UTP, CTP, and GTP not being able to open up double-stranded DNA (DNA-T + DNA-M). The fluorescence intensity of the mixture sensing system was found to be lower than that of the ATP sensing system. Because in the mixture sensing system and the ATP sensing system, the ATP concentration was 753.8 nM and 887.7 nM, respectively. The ATP concentration of the mixture sensing system was 133.9 nM lower than that of the ATP sensing system, which resulted in the mixture sensing system decreased in fluorescence intensity. Therefore, it can be concluded that this sensor has strong specificity and is therefore very suitable for the detection of ATP.

### 3.5. Detection of ATP Concentration in Real Urine Samples

In order to verify the practicability of the method, ATP was added into human urine in order to carry out standard recovery tests. As shown in Table 4, the recovery rate was found to be between 102 and 104.2, and the results indicate that the method is reliable and has great potential for ATP detection in actual urine samples.

## 4. Conclusions

In summary, a label-free, fluorescent, enzyme-free, highly sensitive AND logic gate biosensor was developed for ATP detection. By suppressing the background signal with GO, the sensitivity of the sensor was improved. The sensor was found to have high sensitivity and linearity and was able to detect lower concentrations of ATP with strong selectivity. Therefore, a simple, low-cost, highly sensitive method for the detection of ATP was developed that can be used as a universal detection method for the detection of ions, proteins, and other substances.

## Figures and Tables

**Table 1:** DNA sequences.

Oligonucleotide	Sequence
DNA-T	5′-CGC GCG ACC TGG GGG AGT ATT GCG GAG GAA GGT-3′
DNA-M	5′-ACC TTC CTC CCC AGG T-3′
DNA-M′	5′-ACC TGG GGA GGA AGG T-3′

**Table 2:** A Boolean operation list.

*I1 = (ATP)*	*I2 = (DNA-M′)*	*Output = (ΔF)*
*0*	*0*	*0*
*0*	*1*	*0*
*1*	*0*	*0*
*1*	*1*	*1*

**Table 3:** Comparison of other methods for ATP detection.

Methods	LOD	Linear Range	Correlation Coefficient (R^2^)	Reference
A Universal and Label-free Aptasensor	23.4 nM	0–10 mM	N/A	[33]
An “off-on” Phosphorescent Aptasensor Switch	0.9 nM	2 nM–9 µM	0.995	[34]
Hollow NiFe PBA Derived NiOxFeOy@mC	1.62 fM	8.26 fM–8.26 nM	0.996	[35]
Photoinduced Regeneration	0.5 nM	1 nM–100 μM	0.996	[36]
Mixed Self-assembled Aptamer and Zwitterionic Peptide	0.1 pM	0.1 pM–5 nM	0.9984	[37]
Silver Nanoparticle-decorated Graphene Oxide	5.0 nM	10–850 nM	0.9901	[38]
Peroxidase-like Activity of DNAzyme	2.4 nM	5–230 nM	0.9854	[39]
Unmodified Gold Nanoparticles	0.1 μM	0–5 μM	0.9989	[40]
Oligonucleotide-templated Fluorescent Copper Nanoparticles	500 nM	1–80 mM	0.9901	[41]
Target-induced Conformational Change of Dual-hairpin DNA	1.4 nM	5 nM–1 μM	0.9976	[42]
GO-Based Aptamer Logic Gates	142.6 pM	20–400 nM	0.9943	This work

**Table 4:** Detection of ATP concentration in real urine samples.

	ATP		Proposed	Method	
Samples	Added	Found	Recovery	Standard Deviation (*n* = 3)	Relative Standard Deviation (*n* = 3)
	(nM)	(nM)	(%)	(nM)	(%)
1	100.00	114.368	104.2	7.75	7.4
		102.745			
		95.551			
2	200.00	211.597	102.5	4.95	2.4
		203.427			
		199.758			
3	300.00	308.788	102.0	1.95	0.6
		305.366			
		304.202			
